# Study protocol: a randomised controlled trial investigating the effect of a healthy lifestyle intervention for people with severe mental disorders

**DOI:** 10.1186/1471-2458-11-10

**Published:** 2011-01-05

**Authors:** Amanda Baker, Frances J Kay-Lambkin, Robyn Richmond, Sacha Filia, David Castle, Jill Williams, Terry J Lewin

**Affiliations:** 1 Centre for Brain and Mental Health Research (CBMHR), Faculty of Health, University of Newcastle, Callaghan, NSW, 2308, Australia; 2 National Drug and Alcohol Research Centre (NDARC), University of New South Wales, Sydney, NSW, 2052, Australia; 3 School of Public Health and Community Medicine, University of New South Wales, Sydney, NSW, 2052, Australia; 4 Monash Alfred Psychiatry Research Centre (MAPrc), The Alfred, and School of Psychology and Psychiatry, Monash University, Melbourne, Victoria, 3800, Australia; 5 University of Melbourne and Department of Psychiatry, St Vincent's Hospital, Fitzroy, Victoria, 3065, Australia; 6 UMDNJ-Robert Wood Johnson Medical School, 317 George St, Suite 105, New Brunswick, NJ 08901, USA; 7 Hunter New England Mental Health, PO Box 833, Newcastle, NSW, 2300, Australia

## Abstract

**Background:**

The largest single cause of death among people with severe mental disorders is cardiovascular disease (CVD). The majority of people with schizophrenia and bipolar disorder smoke and many are also overweight, considerably increasing their risk of CVD. Treatment for smoking and other health risk behaviours is often not prioritized among people with severe mental disorders. This protocol describes a study in which we will assess the effectiveness of a healthy lifestyle intervention on smoking and CVD risk and associated health behaviours among people with severe mental disorders.

**Methods/Design:**

250 smokers with a severe mental disorder will be recruited. After completion of a baseline assessment and an initial face-to-face intervention session, participants will be randomly assigned to either a multi-component intervention for smoking cessation and CVD risk reduction or a telephone-based minimal intervention focusing on smoking cessation. Randomisation will be stratified by site (Newcastle, Sydney, Melbourne, Australia), Body Mass Index (BMI) category (normal, overweight, obese) and type of antipsychotic medication (typical, atypical). Participants will receive 8 weekly, 3 fortnightly and 6 monthly sessions delivered face to face (typically 1 hour) or by telephone (typically 10 minutes). Assessments will be conducted by research staff blind to treatment allocation at baseline, 15 weeks, and 12-, 18-, 24-, 30- and 36-months.

**Discussion:**

This study will provide comprehensive data on the effect of a healthy lifestyle intervention on smoking and CVD risk among people with severe mental disorders. If shown to be effective, this intervention can be disseminated to treating clinicians using the treatment manuals.

**Trial registration:**

Australian New Zealand Clinical Trials Registry (ANZCTR) identifier: ACTRN12609001039279

## Background

Cardiovascular disease (CVD) is the largest single cause of death among people with schizophrenia [1] and bipolar disorder [2]. The majority of people with schizophrenia and bipolar disorder smoke [3], considerably increasing their risk of CVD. Many also suffer from obesity, related to inactivity, unhealthy diets and some psychiatric medications [4]. Despite increasing recognition of the widespread impact that smoking and other unhealthy behaviours have on increased morbidity and mortality, treatment is often neglected among people with severe mental disorders. This randomised controlled trial of a Healthy Lifestyles intervention is the first of its kind to address these issues by employing a multi-component healthy lifestyle intervention for smoking and CVD risk behaviours among people with severe mental disorders.

The Healthy Lifestyles intervention was evaluated in a pilot program [5] which showed that setting a variety of goals, and assisting clients to make small steps towards these, can boost motivation and self-confidence to change unhealthy lifestyle behaviour. In that study we developed and piloted a multi-component Healthy Lifestyles intervention aimed at CVD risk reduction and smoking cessation among 43 smokers with severe mental disorders. Primary dependent variables were CVD risk score and smoking. Secondary dependent variables included weight, physical activity, unhealthy eating, substance use, psychiatric symptomatology, treatment retention, general functioning, and quality of life. Significant improvements in the primary dependent variables, CVD risk and smoking, and secondary dependent variables, weight and physical activity were found. There was also an improvement in diet although this did not reach statistical significance. The results of the pilot study suggested that the CVD risk factor intervention was feasible and effective in significantly reducing CVD risk and smoking among people with severe mental disorders. Excellent retention rates (84% completed all sessions), especially among males, attested to the importance and relevance of the intervention for people with psychosis. Participant reports indicated high levels of satisfaction with the program content, with access to nicotine replacement therapy (NRT) that was tailored to their needs, and with the opportunity to target a range of lifestyle factors that they considered important, but had hitherto been neglected. Many participants felt that they would have benefited from a longer intervention to provide scope to modify all the lifestyle issues targeted by the treatment and to consolidate gains. Other research [6] has reported that extended counselling can be significantly more effective than standard treatment, among depressed smokers. Consequently, we embarked upon the current larger-scale randomised controlled trial, with a longer treatment period; it is funded by competitive research grants from the National Health and Medical Research Council of Australia (project IDs 569210 and APP1009351). This trial is registered with the Australian New Zealand Clinical Trials Registry (ACTRN12609001039279).

## Methods/Design

### Study aims

The purpose of the research described here is to test the effectiveness of a multi-component intervention for smoking cessation and CVD risk reduction among people with severe mental disorders. It is hypothesised that the intervention will produce greater, more sustainable improvements in CVD risk and smoking status relative to the control condition at follow-up.

### Study design & setting

**Figure 1:**
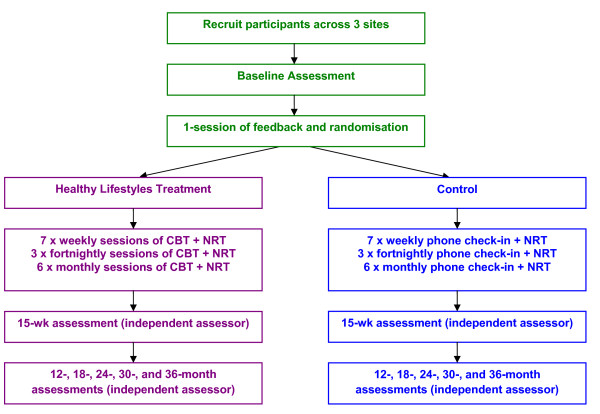
**Study Design**.

This is a prospective randomised controlled comparison study. Figure 1 shows the overall design. After completion of a baseline assessment and an initial face-to-face intervention session, participants will be randomly assigned to either a multi-component intervention for smoking cessation and CVD risk reduction or a telephone-based minimal intervention focusing on smoking cessation. Randomisation will be stratified by site (Newcastle, Sydney, Melbourne, Australia), Body Mass Index (BMI) category (normal, overweight: ≥ 25 and < 30; obese: ≥ 30) and type of antipsychotic medication (typical, atypical). A permuted block randomisation approach will be used so that the distribution of participants across treatment conditions will be maintained regardless of the final sample size. Following completion of the baseline assessment for each participant, the clinicians will be issued with a sealed randomisation envelope (by an independent person) which displays the participant identification code. The envelope will be opened by the participant at the conclusion of the initial session. This research will be conducted in three sites: Centre for Brain and Mental Health Research, University of Newcastle, New South Wales (NSW); School of Public Health, University of NSW, Sydney, NSW; and the Monash Alfred Psychiatry Research Centre (MAPrc), Monash University and The Alfred, Melbourne, Victoria, Australia. The researchers involved are experienced clinicians and scientists. Ethical approval was obtained for this study through the lead site of Hunter New England Human Ethics Committee and at each site.

### Patients

Approximately 250 smokers with a severe mental disorder will be identified from community mental health services, outpatient hospital clinics, psychology and general practices or using self-referral from the general community (e.g. via media advertisements). Written, informed consent will be obtained from each potential volunteer before baseline assessment.

### Inclusion Criteria

1) Age 18 years and over (minimum age level recommended for the use of nicotine replacement therapy, NRT);

2) Diagnosis of a severe mental disorder, as confirmed by the Mini International Neuropsychiatric Interview (MINI; [7]) - schizophrenia spectrum or bipolar disorder;

3) Current smoker (at least 15 cigarettes per day); and

4) Taking antipsychotic medication as prescribed for a period of at least two months, with intention to continue for the duration of the study.

### Exclusion Criteria

1) Non-English speakers;

2) Organic brain diseases; and

3) Medical conditions that would preclude treatment (NRT or overall, e.g. uncontrolled diabetes, pregnancy). Participants will be permitted to access additional treatments outside the proposed study, including psychiatric medication: any such treatments will be recorded at each assessment occasion. Any person who indicates suicidal ideation will be assessed using a standard suicide checklist. Only those persons judged as serious risk for suicide will have their participation suspended and be referred to the relevant psychiatric service. The same protocol will be used for people experiencing an acute phase of their psychotic disorder at any stage in the treatment program.

### Content of the Interventions

Treatment, including NRT, will be provided free to all participants across the face-to-face and phone-based conditions to assist with their attempts at tobacco abstinence and/or reduction. As stated above, all participants will receive an identical first session, after which they will receive four of the 24 weeks supply of NRT. The remaining NRT will be given to participants at weeks 4, 8 and 15. The NRT protocol is flexible and has been described elsewhere [8]. Briefly, participants smoking at least 30 cigarettes per day are eligible to receive double patching in addition to up to 12 × 2 mg lozenges per day, with NRT tapering occurring over the last month of delivery.

Therapists will be psychologists and both the active and control interventions will be guided by manuals (which are available upon request: Baker AL, Kay-Lambkin FJ, Geddes J, Beck A, Sakrouge R, Filia S, Turner A, Clark V: **Healthy Lifestyles intervention therapist manual **and **Healthy Lifestyles intervention telephone manual**. Unpublished manuscripts: University of Newcastle; 2010). Participants will be asked to provide details of their general practitioner (GP), psychiatrist and case worker and to consent to the therapist liaising with these professionals regarding assessment results and treatment progress, management of any acute episodes, and arranging follow-up.

The initial session before randomisation will be conducted face to face with the therapist and will focus on providing feedback to participants regarding their smoking (e.g. level of dependence) and other risk factors for CVD. A case formulation will be developed with the participant regarding their CVD status and unhealthy behaviours, with motivational interviewing (MI) being conducted to help the person consider changes in risk behaviours.

### Healthy Lifestyles Therapist Delivered Intervention (active treatment)

The active treatment protocol focuses on the adoption of more healthy lifestyle choices (see [8] for session by session summary). The initial session will be of 90 minutes duration, followed by seven one hour weekly sessions, three fortnightly hour long sessions and then monthly sessions of one hour duration for six months. A harm reduction focus is an important factor in the engagement and retention of participants, who may present with a range of preparedness to change the lifestyle factors currently impacting on their health and wellbeing. The intervention is designed to encourage smoking cessation and improvements in diet and physical activity, using a combination of MI and cognitive behaviour therapy (CBT) techniques. The initial focus of treatment will be based on the particular CVD risk factor(s), in addition to smoking, considered most problematic by the participant. Therapists will integrate messages and skill development about other CVD risk factors opportunistically. Self-help material will be provided throughout the treatment period, according to the CVD risk factors being discussed in each session.

#### Smoking cessation component

In addition to the provision of NRT that is common to both the Healthy Lifestyle therapist and phone-based conditions, the intervention includes education about the interaction between nicotine and symptomatology, medication and cognition, options for NRT, and examining beliefs regarding the relationship between smoking and symptoms. Despite a harm reduction focus, cessation as the ultimate goal will be encouraged for all participants, and a supportive follow-up telephone call will be made 2-3 days following the initial quit attempt [9]. Nicotine withdrawal symptom severity, cravings to smoke and adverse medication side-effects will be monitored each session.

#### Contingency reinforcement component

In the face-to-face condition, contingent reinforcement will be utilised, as it has been identified as an effective technique for facilitating smoking cessation amongst individuals with severe mental illness [10]. Expired carbon monoxide (CO) will be monitored each week, with positive reinforcement provided in the form of certificates and financial reimbursement (cash and vouchers) when participants meet predetermined criteria for success. The contingency reinforcement schedule will be based on a shaping model. Relative to an abstinent-only model, a shaping model rewards participants for successive reductions in CO readings as well as abstinence. This is in accordance with both abstinence and harm reduction approaches to substance use change. There are several components to this model. Firstly, session-by-session reimbursement is contingent upon demonstrated reductions in expired CO (explained in detail below). Secondly, participants receive a bonus once they meet the CO criterion for a given (set) number of consecutive weeks (e.g. three weeks in a row). Thirdly, an additional 'bonus' is on offer every week for participants who demonstrate abstinence (< 10 ppm expired CO). An advantage of this schedule is that it accounts for individual differences in baseline CO and rates of behaviour change. However, it is anticipated that many participants will be approaching and/or have met the abstinence criterion by the end of the weekly phase of treatment. As such, during the fortnightly and monthly sessions, reinforcement will be contingent upon abstinence only (<10 ppm expired CO). In summary, we will adopt a shaping schedule during the weekly phase of treatment whilst the fortnightly and monthly sessions will provide positive reinforcement only for abstinence.

#### Physical activity component

This component will be integrated with the other components of the Healthy Lifestyles intervention. Specific strategies will be introduced in session 4, with discussion of ways to increase levels of physical activity in everyday life (e.g. taking the stairs rather than the lift) and introduction of a graded walking program with provision of pedometers. Daily pedometer readings have been incorporated into participant monitoring forms, and will also be used to provide objective feedback to treating therapists about the extent of this activity in the day prior to each treatment session. Should participants express a desire to work on their physical activity earlier than session 4, then these strategies will be brought forward in the treatment sequence as required.

#### Dietary and nutrition component

This component will be integrated with the above Healthy Lifestyles strategies with an emphasis on increasing healthy food choices rather than on an 'ideal' caloric intake. Healthy eating habits will initially be discussed in session 7, with food planning and goal setting following in session 8. Specific motivational and CBT-related techniques will include encouraging participants to eat a variety of foods, eating foods that are high in fibre and low in fat, trying to eat five or more servings of fruits and vegetables a day and drinking plenty of water each day, eating regularly, and drinking alcohol within the recommended guidelines for Australia. Participants will be encouraged to consider issues that prevent them from making healthy choices (such as 'non-hungry eating', eating on a budget, cost effective meal plans and planning a shopping list). Finally, medication matters will be addressed, including drug-nutrient interactions and tips for dealing with medication side effects. As with physical activity, nutritional strategies will be brought forward to earlier sessions in the treatment program should participants wish to focus on these issues prior to session 7.

#### Booster sessions

Participants will receive six monthly booster sessions, during which a range of issues can be discussed. These sessions will include relapse prevention, overcoming lapses, review of previous sessions, methods to promote and maintain changes, and NRT tapering.

#### Monitoring

During each treatment session of the Healthy Lifestyle intervention, participants and therapists will complete the following range of formal measurements: side-effects from medication, nicotine withdrawal, weight, cigarettes per day, expired carbon monoxide (CO), NRT use over the past week, and average minutes walking continuously and briskly per week (based on physical activity diary entries).

### Phone-based Condition (control)

In order to orient participants to possible lifestyle changes and in particular, NRT use, those in the phone-based condition will receive an individual 90 minute face-to-face session a week following baseline assessment, as described above. To control for the number of therapist contacts, brief, manualised telephone calls (around 10 minutes) will be conducted with participants in this condition, to 'check in' about smoking and NRT use. Therapists will complete the following formal assessments for each phone session: adverse symptom checklist (antipsychotic medication), nicotine withdrawal and current symptoms of psychosis and mood. Self-report measurements of cigarettes per day, exercise and dietary intake will be taken each week during the phone-based sessions. These phone-based sessions will be made at the same intervals as therapist visits for the Healthy Lifestyles intervention condition (i.e. weekly for eight weeks, fortnightly for three sessions, followed by monthly calls for six months). In place of the phone-based sessions at weeks 4 and 8, participants will attend face to face sessions of 30 minutes duration where NRT is dispensed, and where any problems with NRT or symptomatology are monitored. Biomedical measures (expired CO and weight) will also be taken at these two sessions.

### Treatment Fidelity

Throughout the treatment period, all staff will receive regular weekly clinical supervision. Treatment fidelity will be monitored by delivering the therapy in a consistent fashion, closely adhering to the Healthy Lifestyles and phone-based (control) manuals. In addition, all treatment sessions will be audio recorded. An independent assessor will randomly select a 20% sample of tapes for each therapist, and rate tapes for treatment fidelity. Therapists will also be asked to bring along taped treatment sessions to clinical supervision sessions for discussion among the group.

### Outcome Measures

Outcome measures will be performed at baseline, during treatment (week 15), and at 12-, 18-, 24-, 30- and 36-months after baseline. Baseline assessments will be conducted prior to notification of randomisation status. Post-treatment and follow-up assessments will be conducted by independent assessors who will remain blind to intervention allocation.

All assessment instruments are widely used in mental health and/or tobacco treatment research and practice (see Table 1), and cover the domains hypothesised to be impacted upon by the treatment. CO measures will be taken one hour after arrival to partially control for effects of travelling in traffic, etc. Each participant will be offered up to $20AUD for each assessment, as reimbursement for their out of pocket expenses (e.g. travel).

**Table 1:** Assessment instruments proposed for the current study

Instrument	Initial	15-wk	12-, 18-, 24-, 30- 36-months
*Tobacco Use*			
Opiate Treatment Index (OTI, quantity/frequency) [14]	√	√	√
Point Prevalence and Continuous Abstinence	√	√	√
Readiness to Quit Smoking [15]	√	-	-
Fagerstrom Test for Nicotine Dependence [16]	√	√	√
Expired Carbon Monoxide	√	√	√
Minnesota Nicotine Withdrawal Scale (MNWS-R)[17]	√	√	√
*Physical Activity*			
Readiness to change physical activity [18]	√	-	-
Physical activity screen (past week)	√	√	√
Average minutes walking per week	√	√	√
International Physical Activity Questionnaire [19]	√	√	√
*Dietary Habits*			
Readiness to change eating habits	√	-	-
24-hour recall of eating patterns	√	√	√
*Other CVD Risk Factors*			
OTI- alcohol and cannabis	√	√	√
Beck Depression Inventory - II [20]	√	√	√
Brief Symptom Inventory [21]	√	√	√
Body mass index (height, weight)	√	√	√
Waist-hip ratio (cms)	√	√	√
Auscultatory Blood pressure	√	√	√
Fingerprick Blood Tests (glucose, cholesterol, lipids)	√	√	√
*Treatment and Service Utilisation *[22]	√	√	√
*Diagnosis of psychosis and Symptom Measure*			
Brief Psychiatric Rating Scale [23]	√	√	√
Mini International Neuropsychiatric Interview [22]	√	-	-
*Functioning*			
Global Assessment of Functioning [24]	√	√	√
Medical Outcome Survey SF-36 [25]	√	√	√
Quality of Life Scale [26]	√	√	√
Impact of Weight on Quality of Life Questionnaire [27]	√	√	√
*Therapeutic Alliance & Intervention Satisfaction *[28]	-	√	-

As in the pilot study, the two primary outcome variables will be: (i) overall CVD risk index for participants; and (ii) smoking status; while the secondary dependent variables will include: weight; physical activity; unhealthy eating; substance use; psychiatric symptomatology; treatment retention and treatment alliance; service utilisation; general functioning; and quality of life. The CVD risk index calculation will be performed using the National Vascular Disease Prevention Alliance Absolute Risk Assessment [11] which is based on the Framingham algorithms [12]. Within this index, multiple risk factors of age, gender, systolic blood pressure, cigarette smoking, cholesterol and diabetes are used to predict CVD risk over 5 years [11]. Blood pressure measurements will be taken, as well as a small blood sample by finger prick to measure cholesterol and blood glucose levels. A general health and well-being questionnaire will also be completed by participants to identify lifestyle habits and previous medical history. Smoking status will be determined according to point prevalence abstinence (last 7 days) and continuous abstinence (since quit attempt), both of which will be biochemically validated by a CO reading of ≤10 ppm. Additionally, we will use 50% reduction in cigarettes per day as an indicator of smoking status [13].

We also plan to report the cost of delivering the intervention in real world settings and the cost impacts of the outcomes achieved by calibration of selected instruments used in the study (e.g. Quality of Life Scale, Global Assessment of Functioning) with those achieved in other costing studies.

### Sample size calculation

Prior research conducted by the authors indicates that attrition rates for CBT trials with this sample are, on average, 18% over a 12-month study period following treatment. Thus at 24-month follow-up in the current study (approximately 12 months following the end of treatment), 205 participants will likely remain in the study (102 per treatment condition). Regular contact at every 6 month interval until 36 months post baseline is likely to retain this sample size. This will provide sufficient statistical power (80%) to detect moderate population differences of the order of 0.5 of a standard deviation, using conventional 0.01 level, 2-tailed tests for the primary variables of interest. This is the equivalent of a differential change of approximately 7 cigarettes per day or 13 points on the 100 point CVD risk score, both moderate but clinically useful differences.

### Statistical Analysis

Data coding and analysis will be carried out by the authors using available software packages (e.g. Statistical Package for Social Sciences for Windows). Variables hypothesised to change over time according to treatment allocation will be examined predominantly using generalized linear mixed models, techniques that facilitate management of missing data without imputing values or excluding participants. Chi-square analyses or binary logistic regressions will be performed on categorical outcome variables. Primary outcome measures will typically be analysed in two ways: (1) intention to treat (with study dropouts regarded as continuing smokers and/or with unchanged CVD risk relative to baseline); and (2) analyses performed within the sub-sample of participants who completed the majority of treatment sessions. In addition, comparisons on selected demographic and clinical characteristics will be made between this sub-sample and those who dropped out of treatment, to help detect any biases in outcome measures. Other potential confounders will also be examined (e.g. involvement in additional treatments) and their potential effects modelled in the major analyses (e.g., controlling and not controlling for these variables). As a partial control for the number of statistical tests, the threshold for significance will be set at p < 0.01.

## Discussion

There have been several challenging operational issues in mounting the present trial, which fall into two broad categories: delivering the intended interventions; and recruiting and retaining participants.

### Intervention delivery

The multi-site nature of the trial has required close attention to staff training and supervision. All therapists have prior experience working with people with mental disorders. Manuals for the therapist and telephone delivered conditions are used to guide therapists in each session and detailed protocols have been written for NRT and contingency management components of the intervention. The lead investigator (AB) has trained clinical staff at each site and conducts ongoing weekly group telephone supervision with the therapists. Face to face supervision is also provided separately at each site, with a focus on feedback regarding audiotaped sessions. Therapists usually have to travel to community centres providing mental health treatment, requiring travel time and expenses. While the multi-component nature of the interventions, and our tolerance for additional treatments (outside of the study) are both positive design features, and reflect real-world treatment contexts, they also reduce our capacity to assess the contributions of specific intervention components (or, alternatively, necessitate larger sample sizes, to ensure sufficient numbers of participants who have undertaken particular lifestyle changes).

### Recruitment and retention

Recruiting participants with a severe mental disorder who are prepared to change aspects of their lifestyle requires persistence and flexibility. On the one hand, recruitment into the study has at times been slower than anticipated, with some services referring relatively few people. On the other hand, the stringent entry criteria (e.g., smoking at least 15 cigarettes per day) has meant that some volunteers were necessarily excluded from the study. The assessment battery for the study has been divided into two one and a half hour sessions, due to its length and the need to complete self-report instruments with participants. Engagement in treatment is enhanced by flexible treatment goals and treatment has occasionally been temporarily suspended whilst participants are admitted to hospital or become too unwell to engage in the interventions. Some participants who have poor literacy skills have also been assisted by modification of self-monitoring sheets to include pictures. As the maintenance of behaviour change is of crucial interest, booster sessions and longer-term assessments are highly desirable. Fortunately, additional funding from the National Health and Medical Research Council of Australia has been successfully sought for follow-up over three years.

## Competing interests

The authors declare that they have no competing interests.

## Authors' contributions

All authors contributed to the design of the study and developed the protocol. AB gained ethical approval for the lead site of the trial through the Hunter New England Research Ethics Committee. All authors contributed to manuscript preparation. All authors approved the final manuscript for submission.

## Pre-publication history

The pre-publication history for this paper can be accessed here:

http://www.biomedcentral.com/1471-2458/11/10/prepub
